# Predicting Anxiety in Routine Palliative Care Using Bayesian-Inspired Association Rule Mining

**DOI:** 10.3389/fdgth.2021.724049

**Published:** 2021-08-25

**Authors:** Oliver Haas, Luis Ignacio Lopera Gonzalez, Sonja Hofmann, Christoph Ostgathe, Andreas Maier, Eva Rothgang, Oliver Amft, Tobias Steigleder

**Affiliations:** ^1^Department of Industrial Engineering and Health, Institute of Medical Engineering, Technical University Amberg-Weiden, Weiden, Germany; ^2^Pattern Recognition Lab, Department of Computer Science, Friedrich-Alexander University, Erlangen-Nürnberg, Germany; ^3^Chair of Digital Health, Friedrich-Alexander University, Erlangen-Nürnberg, Germany; ^4^Department of Palliative Medicine, Comprehensive Cancer Center Erlangen-EMN, Friedrich-Alexander-University, Erlangen-Nürnberg, Germany

**Keywords:** anxiety, machine learning, association rule mining, palliative care, routine data, questionnaire

## Abstract

We propose a novel knowledge extraction method based on Bayesian-inspired association rule mining to classify anxiety in heterogeneous, routinely collected data from 9,924 palliative patients. The method extracts association rules mined using lift and local support as selection criteria. The extracted rules are used to assess the maximum evidence supporting and rejecting anxiety for each patient in the test set. We evaluated the predictive accuracy by calculating the area under the receiver operating characteristic curve (AUC). The evaluation produced an AUC of 0.89 and a set of 55 atomic rules with one item in the premise and the conclusion, respectively. The selected rules include variables like pain, nausea, and various medications. Our method outperforms the previous state of the art (AUC = 0.72). We analyzed the relevance and novelty of the mined rules. Palliative experts were asked about the correlation between variables in the data set and anxiety. By comparing expert answers with the retrieved rules, we grouped rules into expected and unexpected ones and found several rules for which experts' opinions and the data-backed rules differ, most notably with the patients' sex. The proposed method offers a novel way to predict anxiety in palliative settings using routinely collected data with an explainable and effective model based on Bayesian-inspired association rule mining. The extracted rules give further insight into potential knowledge gaps in the palliative care field.

## 1. Introduction

A major focus of palliative care is the improvement of quality of life (QoL) of patients suffering from a life-threatening illness by managing their symptoms (1). A variety of different symptoms can have a diminishing effect on the QoL of those affected (2–5). Psychological symptoms and their influence on the QoL of patients are also intensively investigated (5–7). These symptoms often remain underestimated, unrecognized or are not treated, contributing decisively to the patients' suffering (8–10). Therefore, it is crucial to identify psychological distress in patients with terminal illness (11). With regard to anxiety, the German national S3 guideline on Palliative Care in Cancer Patients emphasizes an “early systematic query/recording and documentation of anxiety” (12). The presence of anxiety should be “actively and regularly assessed” and recorded within the framework of the basic palliative care assessment (12).

Various studies examine instruments for the assessment of mental symptoms (6, 8, 13). In the application of these instruments, significant differences in the published prevalence values are striking (8, 13–16). Using a meta-analysis, Mitchell et al. (6) examined the prevalence values of psychiatric disorders in adult cancer patients in oncological, hematological, or palliative care structures with data from interviews in 4,007 palliative care patients in 24 studies from 7 countries. Prevalence were as follows: depression (minor or major depression or dysthymia) 24.6% (range: 17.5%-32.4%), for clinical depression or adjustment disorder 24.7% (range: 20.8–28.8%) and for depression, adjustment disorder or anxiety 29.0% (range: 10.1–52.9%) (6). This data is based on comprehensive and time-consuming validated interviews, which cannot be implemented broadly due to restrictions in both time and expertise (17). In addition, many seriously ill patients were unable to maintain sufficient attention for the duration of a diagnostic interview (8). So alternative options, which are more applicable, have to be evaluated. In a review, Hotopf et al. (8) analyzed a total of 46 studies and 4 case reports on the prevalence of depression and anxiety in patients with advanced diseases and on the most effective detection strategies. Different methods for assessing symptoms were examined: (1) assessment by clinical staff (e.g., doctors, nurses) (2) single-item questions (3) questionnaires (4) diagnostic interviews. In 10 studies, the assessment of symptoms was carried out using diagnostic interviews with a prevalence from 5.6 to 32% due to the aforementioned restrictions. Self-assessment tools (Patient-Reported Outcome Measures, PROMs) are often used in the palliative care setting to determine symptom burden (18, 19). However, validated PROMs as the Hospital Anxiety and Depression Scale (HADS) (20) are in conflict in the context of palliative patients as key indicators can derive from both depression and somatic aspects of the underlying disease (17, 21).

Brief screening scales are another option to identify anxiety with comparatively little burden for the patients (17, 22). Brief screening scales consist of one, two, or three questions asked of the patient, for screening for depression in patients with advanced illness. However, the reliability of short screening scales for the assessment of depression in palliative care patients is unclear (23, 24) and anxiety assessments using short screening scales in patients with life-limiting illnesses is much sparser, showing a low reliability (25). As a result, screening tools may support the early recognition of psychological distress (17, 22), but the reliability is inadequate (26).

Additionally, informal exploration to assess psychological distress in palliative care patients by members of the multi-professional palliative care team can help to identify psychological distress (27, 28). Healthcare professionals should ask open questions about patients' mood, signaling to those affected that psychological symptoms are considered normal in the context of a serious illness and making patients feel less stigmatized and open about sharing depressive symptoms and anxiety (17, 26). To make this feasible, medical staff need to be sensitized and trained to assess psychological symptoms (11, 29). Some symptoms may overlap with those of cancer (e.g., fatigue, listlessness, sleep disturbance, loss of appetite) (30) and informal exploration remains uncertain regarding reliability (31–33), which often complicates exploration.

As palliative care views the patient comprehensively in various dimensions (somatic, social, psychological, and spiritual), previous studies explored classical statistic methods to asses whether other non-psychological symptoms may point to the occurrence or prevalence of psychological symptoms (34). They found that symptoms might occur in clusters, opening the possibility to asses symptom clusters, which are evaluated more reliably, to help identify psychological distress. Comprehensive data sets of patients need to be analyzed to enable a timely therapeutic intervention, which is currently unfeasible in the face of the daily clinical routine (35).

Association rule mining (ARM) is anticipated to identify symptoms that lead or support anxiety in palliative patients. In data mining, association rules are often used to detect frequent patterns in transaction data (36). Association rules are patterns of the form *A* ⇒ *B*, where *A* and *B* are sets of items that occur in the transactions. One could think of customers' shopping baskets in a supermarket as transactions. One association rule could be {bread, butter} ⇒ {eggs}, indicating that customers who buy bread and butter will likely also buy eggs. The sets that occur in the rule are called *item sets*, and their elements *items*. The quality of rules is often measured using *support*, which is the percentage of observations in the data set that contain the item set of interest. If, for example, 66% of all customers in our supermarket buy bread as well as butter, then supp({bread, butter}) is 66%.

The conventional ARM methodology uses *support* as the primary rule selection criteria. Support's downward closure property helps reduce the search space for possible rules (37). However, rule support suffers from dilution as new observations are added (38). In medicine and other fields, frequent patterns can be misleading, as the outcome of interest may be very rare. Therefore, other metrics have been used to mine for association instead of frequency (39). In contrast to support, which considers the whole data set, *local support* (40) only takes observations into account where the outcome of interest occurs. Equation (1) shows local support's formal definition.


(1) $$\begin{array}{l} \text{lsupp}(A\Rightarrow B)=\frac{\text{supp}(A\cup B)}{\text{supp}(B)}. \end{array}$$

As an example, a local support of 50% for a rule *A* ⇒ *B* means that *A* occurs in 50% of the observations in which *B* occurs. In the probabilistic view, local support corresponds to *P*(*A* ∣ *B*), which is the probability of observing *A* given that we observed *B*.

Another frequently used rules selection criterion in ARM is lift (41). Equation (2) illustrates lift's definition for two item sets *A* and *B*. One can think of supp(*X*) for a given set *X* as an approximation of the probability *P*(*X*) of observing all items from set *X* in a data set transaction. Thus, the probabilistic interpretation provides an alternative explanation of why lift can be used to measure the predictive value of a rule. Specifically, using the ratio *P*(*B* ∣ *A*)/*P*(*B*), we can quantify how information on *A* improves our prediction of the occurrence of *B*.


(2) $$\begin{array}{l} \text{lift}(A\Rightarrow B)=\frac{\text{supp}(A\cup B)}{\text{supp}(A)\cdot \text{ supp}(B)}. \end{array}$$

In this work, we used the increasing belief criterion (IBC) (38) to overcome support's shortcomings for rare rule extraction and robustness against dilution. IBC defines the belief of a rule as the probability of observing the premise given the conclusion after *k* observations following a Bayesian belief update process. In particular, given a rule *A* ⇒ *B*, belief is the probability of *P*(*B*∣*A*)_1_ evaluated at the first rule observation in the data set. Furthermore, IBC states that a rule is considered useful if the belief increase when we take more observations into consideration. Using the recursive definition of the Bayes' theorem, Equation (3) illustrates IBC's definition, where *k* is the number of rule observations in the data set. *P*(*X*)_*k*_ denotes the empirical estimation of *P*(*X*) after the *k*-th observation of the rule *A* ⇒ *B*. The selection criterion of increasing belief is formulated as *B*(*A* ⇒ *B*)_*k*_ ≥ *B*(*A* ⇒ *B*)_*k*−1_.


(3) $$\begin{array}{l} B{\left(A\Rightarrow B\right)}_{1}=P{\left(B|A\right)}_{1}=\frac{P{\left(A|B\right)}_{1}P{\left(B\right)}_{1}}{P{\left(A\right)}_{1}}\\ B{\left(A\Rightarrow B\right)}_{k}=\frac{P{\left(A|B\right)}_{k}B{\left(A\Rightarrow B\right)}_{k-1}}{P{\left(A\right)}_{k}} \end{array}$$

The aim of this work is to develop a novel prediction model for anxiety in palliative patients based on the HOspice and Palliative care Evaluation (HOPE) data set. To make this model transparent and easy to interpret, we created an anxiety prediction model based on association rules and IBC. This work provides the following contributions: (1) We illustrate the IBC approach to knowledge extraction and its correspondence to lift. We describe rule selection criteria and how we construct a prediction model to classify anxiety from the extracted rules. (2) We apply our rule mining and prediction approach to the public HOPE data set and derive classification performance. (3) We compare the mined rules to independent ratings of palliative care experts and discuss agreements and divergences.

## 2. Method

The problem of detecting anxiety using ARM can be generalized to a binary classification problem, where the model provides evidence in favor a data record belonging to the class anxiety or the class no-anxiety. Our proposed approach uses ARM and IBC to extract rules using two parameters: maximal rule length *r*_*l*_ and local support threshold θ. The resulting rules are categorized into yes- and no-rules, depending on whether the outcome in the right-hand side was anxiety-yes or -no. We apply the mined rule set as a prediction model to classify anxiety. For each new data record, we select the rules whose premise matches the record's variable values. The prediction model classifies the record based on the difference between aggregated lift of yes- and no-rules. A decision boundary parameter *d* is used to moderate the prediction model's response.

We implemented both the knowledge extraction process and the classification based on the mined rules from the ground up in the C# programming language.

### 2.1. Knowledge Extraction

We analyzed the recursive part of IBC's definition as illustrated in Equation (4).


(4) $$\begin{array}{l} \;B{\left(A\Rightarrow B\right)}_{k}\geq B{\left(A\Rightarrow B\right)}_{k-1}\\ \frac{P{\left(B|A\right)}_{k}B{\left(A\Rightarrow B\right)}_{k-1}}{P{\left(A\right)}_{k}}\geq B{\left(A\Rightarrow B\right)}_{k-1}\\ \;\frac{P{\left(B|A\right)}_{k}}{P{\left(A\right)}_{k}}\geq 1 \end{array}$$

By applying Bayes' theorem to the left side numerator in Equation (4), we observe that lift(*A*)_*k*_ ≥ 1 and lsupp(*A*)_*k*_ > *P*(*A*)_*k*_ emerge as criteria as illustrated in Equation (5).


(5) $$\begin{array}{l} \frac{P{\left(A\cap B\right)}_{k}}{P{\left(A\right)}_{k}P{\left(B\right)}_{k}}\geq 1\Leftrightarrow \text{lift}{\left(A\right)}_{k}\geq 1\\ \frac{P{\left(A\cap B\right)}_{k}}{P{\left(B\right)}_{k}}\geq P{\left(A\right)}_{k}\Leftrightarrow \text{lsupp}{\left(A\right)}_{k}\geq P{\left(A\right)}_{k} \end{array}$$

Based on these findings, we constructed a mining framework where support was replaced by lift(*A*) ≥ 1 as primary rule selection criterion. Initial experiments revealed that the criterion lift(*A*) ≥ 1 resulted in many rules with inadequate predictive performance. Thus, we introduced θ, a threshold on local support, as secondary rule selection criterion to filter out rules.

Furthermore, we consider the maximal rule length parameter *r*_*l*_, which determines the maximum number of items in the premise. For example, a maximal rule length of two means that only rules with one or two items in the premise were used and rules of the form {*x*1, *x*2, *x*3} ⇒ *B* were excluded. To search for complex rules, i.e., *r*_*l*_ greater than one, we use the downward closure property of local support and support. The downward closure property states that all item sets that contain an item set with a local support or support below some threshold also have a local support or support below the threshold. We can thus ignore all supersets of item sets with a local support or support below the threshold. The downward closure property does not hold for lift and was thus enforced, e.g., a rule {*x*1, *x*2} ⇒ {*y*} was only kept if this rule as well as the two sub-rules {*x*1} ⇒ {*y*} and {*x*2} ⇒ {*y*} each had lift ≥ 1.

The filtering steps mine the sets of rules *R*_yes_ (if the rules conclusion is that anxiety is present) and *R*_no_ (if anxiety is not present) of yes- and no-rules, for which every sub-rule as well as the rule itself have a lift ≥ 1, and which have a local support or support of at least θ, i.e.,


(6) $$\begin{array}{l} R_{\text{yes}}=\{r=A\Rightarrow \{\text{yes}\}|\text{lsupp}(r)\geq \theta ,\forall A^{\prime }\subset A:\text{lift}(A^{\prime }\Rightarrow \{\text{yes}\})\geq 1\},\\ R_{\text{no}}=\{r=A\Rightarrow \{\text{no}\}|\text{lsupp}(r)\geq \theta ,\forall A^{\prime }\subset A:\text{lift}(A^{\prime }\Rightarrow \{\text{no}\})\geq 1\}, \end{array}$$

### 2.2. Prediction Model

The prediction was based on both the no-rules *R*_no_ and the yes-rules *R*_yes_. For any patient *p*, both rule sets were evaluated separately with the following steps. First, only the rules that apply to the patient were used, i.e., rules in which the patient's data variables matched the rule premise. As a result, we obtained two rule sets *R*_yes,*p*_ and *R*_no,*p*_, which contain all the yes- and no-rules that apply to patient *p*. Second, the lift of all rules in each set *R*_yes,*p*_ and *R*_no,*p*_ was aggregated using an aggregation function α.

The difference between the two aggregated lift values were derived and binarized by applying an empirical decision boundary *d*. The algorithm's decision function *f*(*p*) is shown in Equation (7), where α(*R*_yes,*p*_) and α(*R*_no,*p*_) are the respective aggregated lift values of yes- and no-rules that apply to patient *p*.


(7) $$\begin{array}{l} f(p)=\{\begin{array}{ll} \text{anxiety,} & \text{ if }\alpha (R_{\text{yes},p})-\alpha (R_{\text{no},p})\geq d,\\ \text{no anxiety,} & \text{ if }\alpha (R_{\text{yes},p})-\alpha (R_{\text{no},p})<d. \end{array} \end{array}$$

## 3. Evaluation

The present study was reviewed by the ethics board of the Friedrich-Alexander-Universität Erlangen-Nürnberg.

### 3.1. Palliative Patient Data

We used data from the “Hospiz- und Palliativ-Erfassung” (HOspice and Palliative Evaluation, HOPE) project (42). HOPE offers a standardized questionnaire that can be used by hospice and palliative care units across Germany to document patient status. It includes items on general information, medication, problems the patient experiences, and the organization of care.

The HOPE data set was previously prepared and analyzed (34). The HOPE data set contains information on 9,924 patients in stationary care centers throughout Germany during a 3-month documentation period between 2007 and 2011. In total 40 individual variables with different values were acquired. Among the included patients, 5,149 (51.9%) were female, 4,694 (47.3%) were male, and for the remaining 81 patients (0.8%), no gender was recorded. No or mild anxiety was reported for 6,127 patients (61.7%), with the remaining 3,797 patients (38.3%) having moderate or severe anxiety.

In this work, we consider each patient as one observation. Items were considered as pairs of variable name and value, e.g., a patient, who had scored “1” in the variable “laxatives,” was considered having an item “laxatives=1.”

In the original data set, anxiety was encoded with four different levels: none, mild, moderate, and severe. To keep results comparable with Hofmann et al. (34), anxiety was dichotomized as “no anxiety” (anxiety = 0, encompassing none and mild) and “anxiety” (anxiety = 1, encompassing moderate and severe). Furthermore, this dichotomization derives from clinical practice where none or mild anxiety means that no further treatment is required and moderate or severe anxiety prompts a clinician's reaction.

### 3.2. Prediction Validation Methods

To mine the no- and the yes-rules, 66.6% (around 6,600) of the patients in the data set were used. The remaining 33.3% (around 3,300) were used to test the method.

We ran two different sets of experiments to compare the effect of the support and local support metrics. In the first set of experiments, we used lift as the primary metric and local support as the secondary metric. We fixed lift≥1.0. The local support threshold was varied between 0.0 ≤ θ ≤ 0.5 in increments of 0.1. The maximal length of rules *r*_*l*_ was ranged between 1 ≤ *r*_*l*_ ≤ 3. Each experiment was repeated with 10 times with one of three aggregation functions: average (α_avg_), maximum (α_max_), and sum of lifts (α_sum_), as defined in Equation (8) for *R*_yes_ rules.


(8) $$\begin{array}{l} \alpha_{\text{avg}}(R_{\text{yes},p})=\frac{1}{\left|R_{\text{yes},p}\right|}\sum_{r\in R_{\text{yes},p}}\text{lift}(r),\\ \alpha_{\text{max}}(R_{\text{yes},p})=\underset{r\in R_{\text{yes},p}}{\max}\text{lift}(r),\\ \alpha_{\text{sum}}(R_{\text{yes},p})=\sum_{r\in R_{\text{yes},p}}\text{lift}(r), \end{array}$$

To compare performance with well-established ARM metrics, we replaced local support with support, and repeated the experiment sets using a support threshold ranged between 0.0 ≤ θ ≤ 0.5 in increments of 0.1. The range of *r*_*l*_, aggregation functions and lift threshold values remained unchanged.

The different experiments were evaluated using the area under the receiver operating characteristic curve (AUC) (43). The decision boundary parameter *d* was set using the ROC curves used for AUC calculation. We selected a configuration with best performance on the test data and compared it with the state of the art.

### 3.3. Palliative Professional Assessment

We designed a questionnaire to compare the knowledge extracted by IBC mined rules with the opinion of palliative care professionals. For each item in the HOPE data set, palliative professionals were asked if they expected the correlation between the variable and anxiety to be highly negative (−2), mildly negative (−1), non-existent (0), mildly positive (1), or highly positive (2). Due to the 20 different possible values for the variable *group-of-diagnosis*, the question was stated as follows: “The patient's *group-of-diagnosis*is” (a) “correlated in some diagnosis groups,” (b) “correlated in all diagnosis groups,” or (c) “uncorrelated.” Moreover, the professionals were asked to describe additional variables that they consider correlated with anxiety in palliative care patients. The questionnaire was sent to active palliative care physicians.

## 4. Results

We used the Accord framework (44), version 3.8.0, to compute the AUC values. All further analysis was done in the statistical programming language R (45), version 4.1.0, with the tidyverse packages, version 1.3.1 (46).

### 4.1. Anxiety Classification

**Figure 1:**
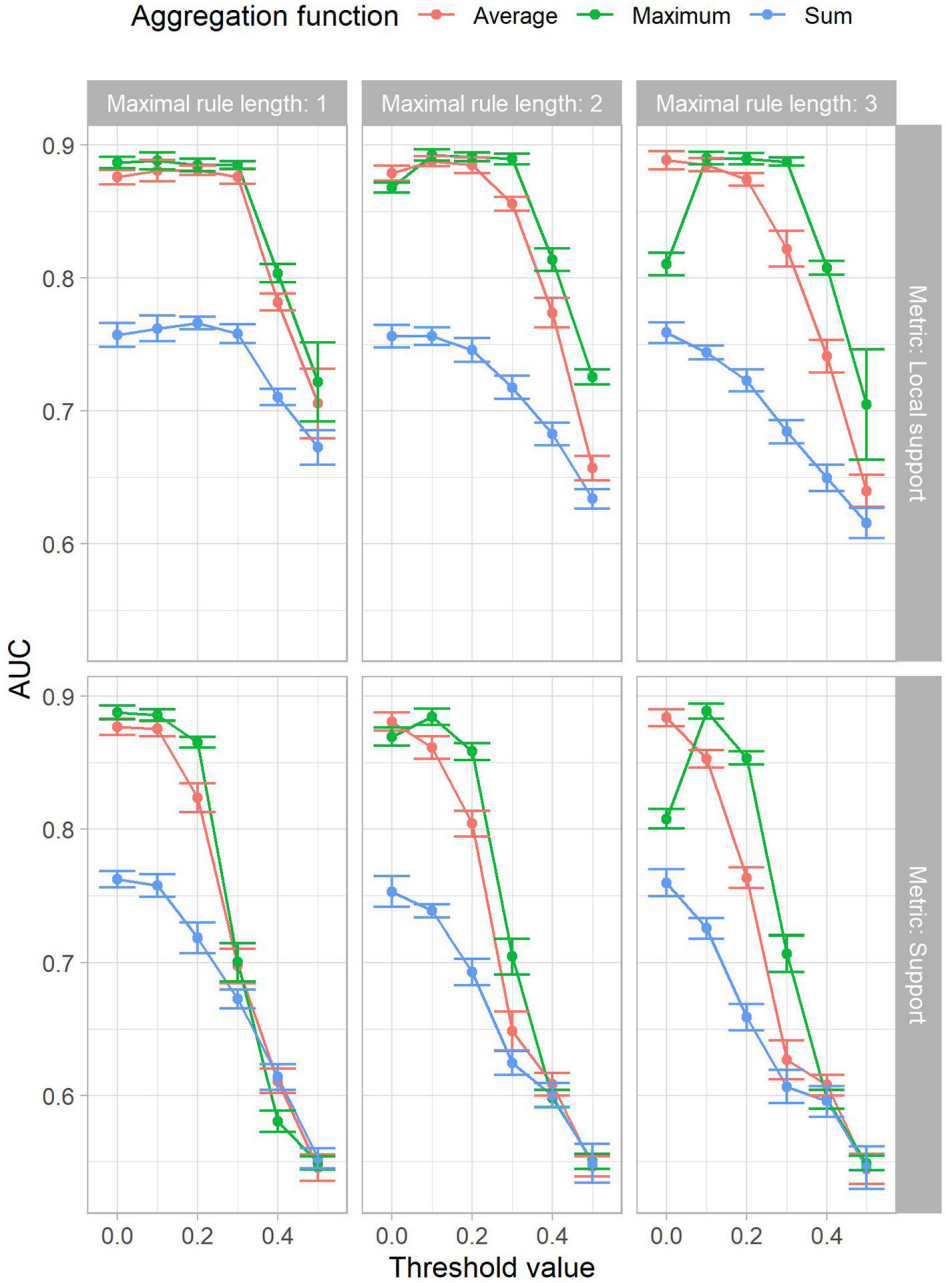
The mean AUC achieved in the experiments by maximal rule length (horizontal facets), local support or support (x-axis in the top resp. bottom facets) and aggregation function (color). The standard deviation is shown by the size of the error bars.

We computed the AUC by varying the decision boundary and calculating the prediction model's sensitivity and specificity on the test set. Figure 1 shows the AUC results for all experiment configurations. For local support, the average AUC over 10 experiment runs ranged from 0.61 to 0.89. In most cases, the local support filter increases the AUC up to a local support threshold value of 0.3, after which the AUC decreases. In comparison, using the support filter yielded an AUC range between 0.54 and 0.89, where the AUC starts decreasing after a threshold value of 0.1. Therefore, the local support filter appears more robust than support across threshold changes. Thus, it is preferable to use local support.

The results show that the max aggregation α_max_ outperforms all other aggregation function variants. In particular, α_max_ has a more consistent performance across threshold values and maximal rule lengths. Furthermore, using a threshold value of 0.0 is equivalent to disabling the second criterion filter, as all rules will pass. Therefore, the lift criterion, i.e., IBC, in combination with the classification function *f*(*p*) and the aggregation function α_max_ can yield adequate AUC.

Atomic rules, i.e., *r*_*l*_ = 1, resulted in larger AUC compared to longer ones, i.e., *r*_*l*_ > 1. As *r*_*l*_ increases, the AUC drops. Local support recovers performance for some aggregation functions, while it displays the stable region until the 0.3 threshold value. Nevertheless, the variation between experiments with increased maximal rule lengths still exhibit less AUC.

**Figure 2:**
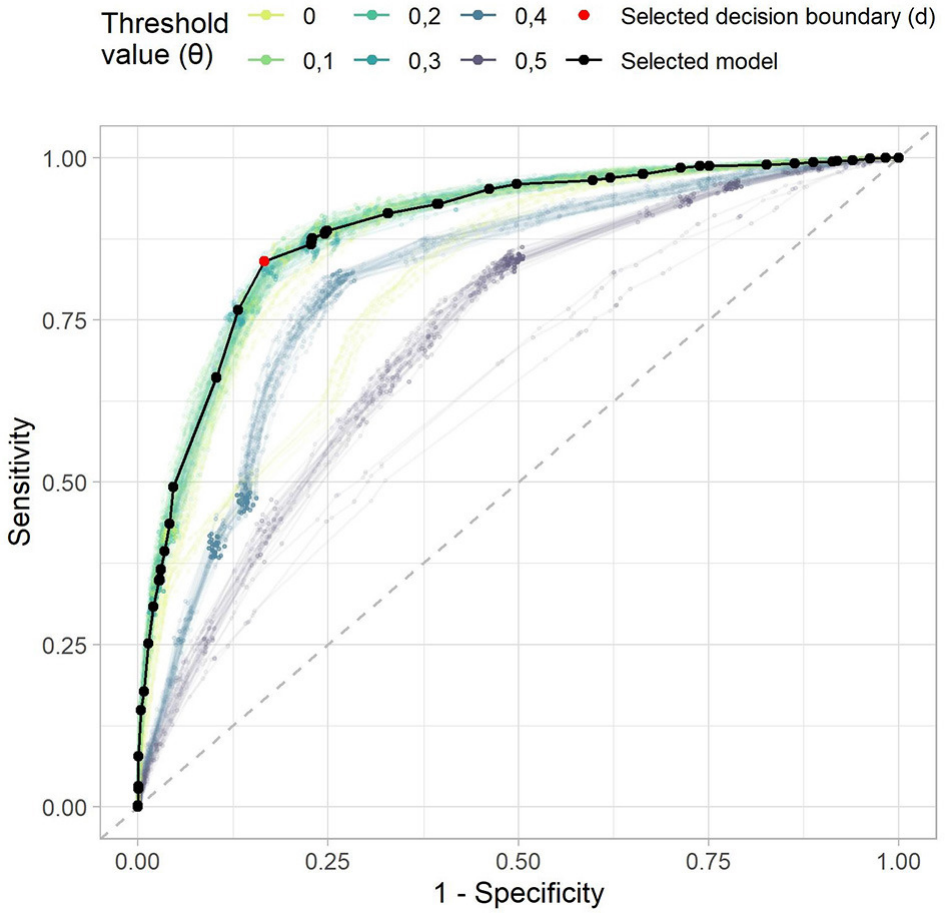
The receiver operating characteristic of the example rule sets. The dashed gray line denotes a random predictor. The model that we selected for further analysis is shown in black, the selected decision boundary *d* in red. Each point denotes one possible decision boundary, while the x-axis denotes one minus the specificity, and the y-axis the sensitivity of the model given this decision boundary. The color lines indicate the curves of all other models with the maximum aggregation function.

Increased thresholds for support and local support, in combination with a low maximal rule length, i.e., *r*_*l*_ = 1, led to faster execution times and less rules in the model. Furthermore, we observed that the performance is comparable among multiple parameter configurations. Therefore, a model with as few rules as possible, while still retaining the high AUC, was preferred. Using a decision boundary *d* that maximizes the sum of specificity and sensitivity, a top AUC of 0.89 was obtained for the parameter configuration with local support of θ = 0.3, a maximal rule length of *r*_*l*_ = 1, the maximum function α_max_ for aggregation, and a decision boundary value *d* = 0.33. The resulting confusion matrix can be seen in Table 1. The selected point in the ROC curve had a specificity of 83.4% and a sensitivity of 84.0%, other performance metrics are shown in Table 2. The top AUC performance configuration returned 29 no-rules and 26 yes-rules. The corresponding receiver operator characteristic (ROC) in comparison to other parameter configurations can be seen in Figure 2. The corresponding rule set premises included 37 different variables, i.e., from the 40 HOPE variables only the target variable anxiety as well as variables age and group diagnosis were left out. The yes-rules included 23 of these variables, while the no-rules included 27 variables. The rules included in the prediction model can be found in the Supplementary Material.

**Table 1:** The confusion matrix of our prediction model.

	**Anxiety**	**No anxiety**
Predicted anxiety	1,017	305
Predicted no anxiety	211	1,624

**Table 2:** Various metrics that describe our prediction model's performance.

**Metric**	**Value (%)**
Accuracy	84
Sensitivity	83
Specificity	84
Positive predictive value	77
Negative predictive value	89

### 4.2. Interpretability of Mined Rules

An example patient record is shown in Table 3. Out of all the rules, eight yes-rules and 20 no-rules apply to the example record. The corresponding rules of the example record are shown in Table 4. As the maximum yes-lift [α_max_(*R*_yes,*p*_) = 1.07] is lower than the no-lift [α_max_(*R*_no,*p*_) = 1.56], the difference score according to Equation (7) is 1.07−1.56 = −0.49. With the decision boundary *d* = 0.33, Equation (7) yields *f*(*p*) = no anxiety. Thus, the algorithm predicts that the patient does not suffer from anxiety, which is correct according to the HOPE-reported variable anxiety = 0.

**Table 3:** One example record from the data set.

**Variable**	**Value**	**Variable**	**Value**
Advance_dir	0	Laxatives	1
Age	0	Level_nursing_care_support	5
Antibiotics	−1	Living_situation	3
Antidepressants	−1	Loss_appetite	0
Antiemetics	−1	Nausea	0
Anxiety	0	Neuroleptics	−1
Assistance_adl	1	Non_opioids	−1
Brain_metastases	1	organization_of_care	0
Cardiacs	−1	other_drugs	−1
Co_analgetics	−1	Pain	1
Constipation	0	Pal_care_service	4
Corticosteroids	1	Sedatives_anxiolytics	−1
Disorientation_confusion	1	Tension	0
Diuretics	−1	Tiredness	1
Dyspnea	0	Type_disease	1
ECOG	1	Vomiting	0
Feeling_depressed	0	Weakness	2
Gastric_protection	1	WHO_2	−1
Gender	2	WHO_3	−1
Group_diagnosis	1	Wound_care	0

**Table 4:** All the rules that apply to the record described in Table 3.

**Premise**	**Anxiety**	**Lift**	**Local supp**.	**Supp**.	**Supp. in test set**
Advance_dir = 0	No	1.00	0.69	0.42	0.42
Antibiotics = −1	No	1.01	0.87	0.53	0.53
Antidepressants = −1	No	1.05	0.85	0.52	0.52
Antiemetics = −1	No	1.03	0.69	0.42	0.44
Cardiacs = −1	Yes	1.02	0.71	0.28	0.28
Co_analgetics = −1	No	1.03	0.79	0.48	0.50
Constipation = 0	No	1.10	0.42	0.26	0.27
Corticosteroids = 1	Yes	**1.07**	0.32	0.13	0.12
Diuretics = −1	Yes	1.01	0.73	0.28	0.27
Dyspnea = 0	No	1.14	0.51	0.31	0.33
Feeling_depressed = 0	No	1.37	0.51	0.31	0.32
Gastric_protection = 1	Yes	1.03	0.57	0.22	0.21
Gender = 2	No	1.05	0.49	0.30	0.32
Laxatives = 1	Yes	1.06	0.39	0.15	0.15
Living_situation = 3	Yes	1.03	0.68	0.27	0.26
Nausea = 0	No	1.11	0.54	0.33	0.34
Neuroleptics = −1	No	1.01	0.91	0.55	0.57
Non_opioids = −1	No	1.00	0.50	0.31	0.32
Organization_of_care = 0	No	1.16	0.38	0.23	0.24
Other_drugs = −1	Yes	1.01	0.79	0.31	0.29
Sedatives_anxiolytics = −1	No	1.09	0.79	0.48	0.50
Tension = 0	No	**1.56**	0.37	0.23	0.24
Type_disease = 1	No	1.00	0.89	0.55	0.57
Vomiting = 0	No	1.06	0.74	0.45	0.47
Weakness = 2	No	1.07	0.32	0.20	0.22
WHO_2 = −1	Yes	1.00	0.92	0.36	0.34
WHO_3 = −1	No	1.09	0.43	0.26	0.28
Wound_care = 0	No	1.04	0.63	0.39	0.42

*The maximal lifts for yes and no are in bold*.

To explain the rule set meaning verbally, only the maximum lift rules “tension = 0” and “corticosteroids = 1” were considered, as the corresponding aggregation function α_max_ yielded top AUC performance. In textual form, this reasoning can be explained as “The patient takes corticosteroids, which is associated with having anxiety with a lift of 1.07, which is the highest association with anxiety this patient shows. On the other hand, the highest association with not having anxiety is that they experience no tension, which has a lift of 1.57. As the no-lift outweighs the yes-lift by more than 0.33, the algorithm's prediction is that the patient does not suffer from anxiety.”

### 4.3. Palliative Professional Assessment

**Figure 3:**
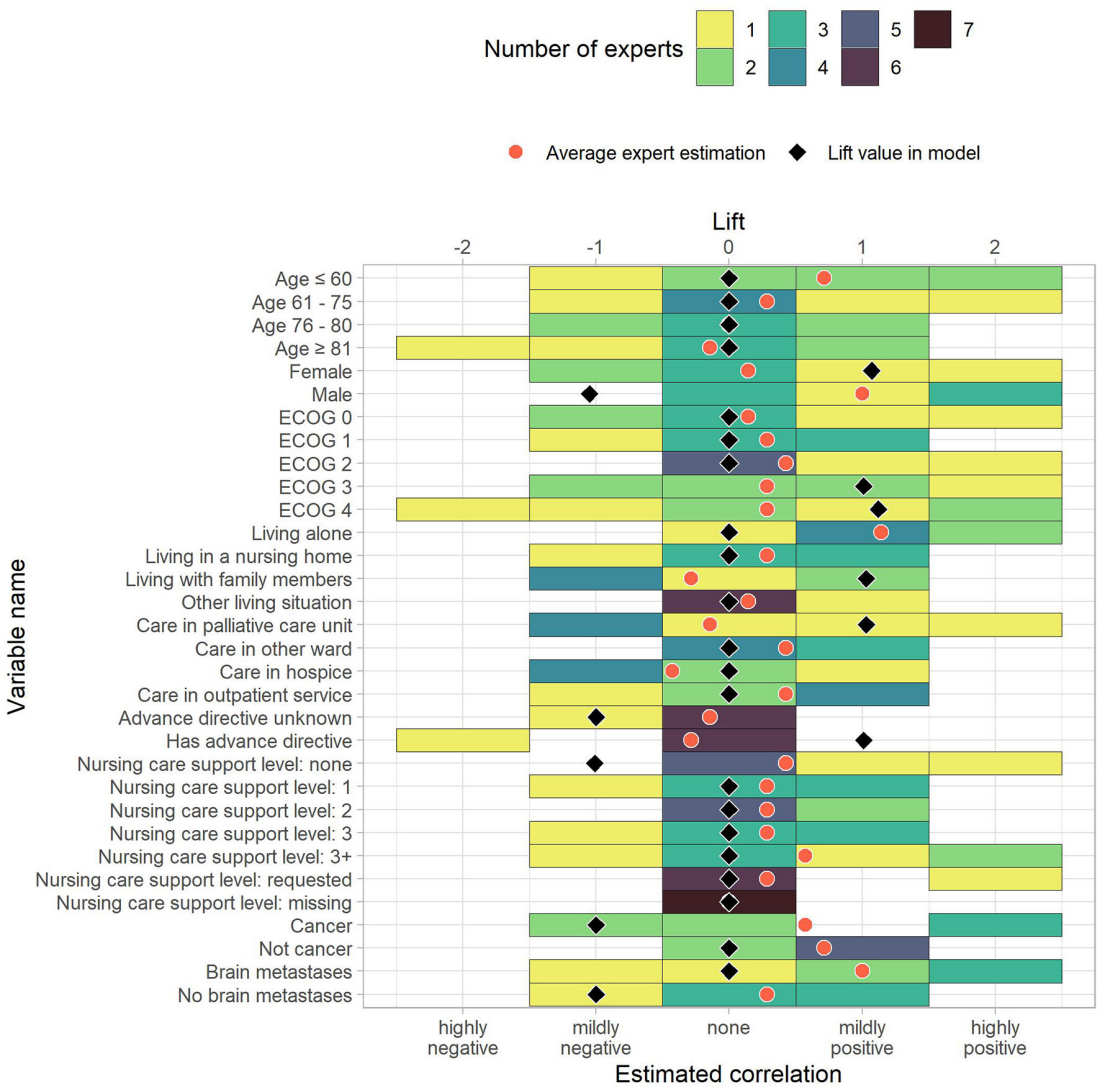
The estimated correlations of HOPE variables to anxiety for demographic and administrative data as well as diagnoses. The color indicates how many experts chose the corresponding option. The black diamond indicate the lift value of the corresponding variable in the model, where 0 stands for “not part of the model” and negative values correspond to the lift of no-rules. The red dot is the average expert estimation.

**Figure 4:**
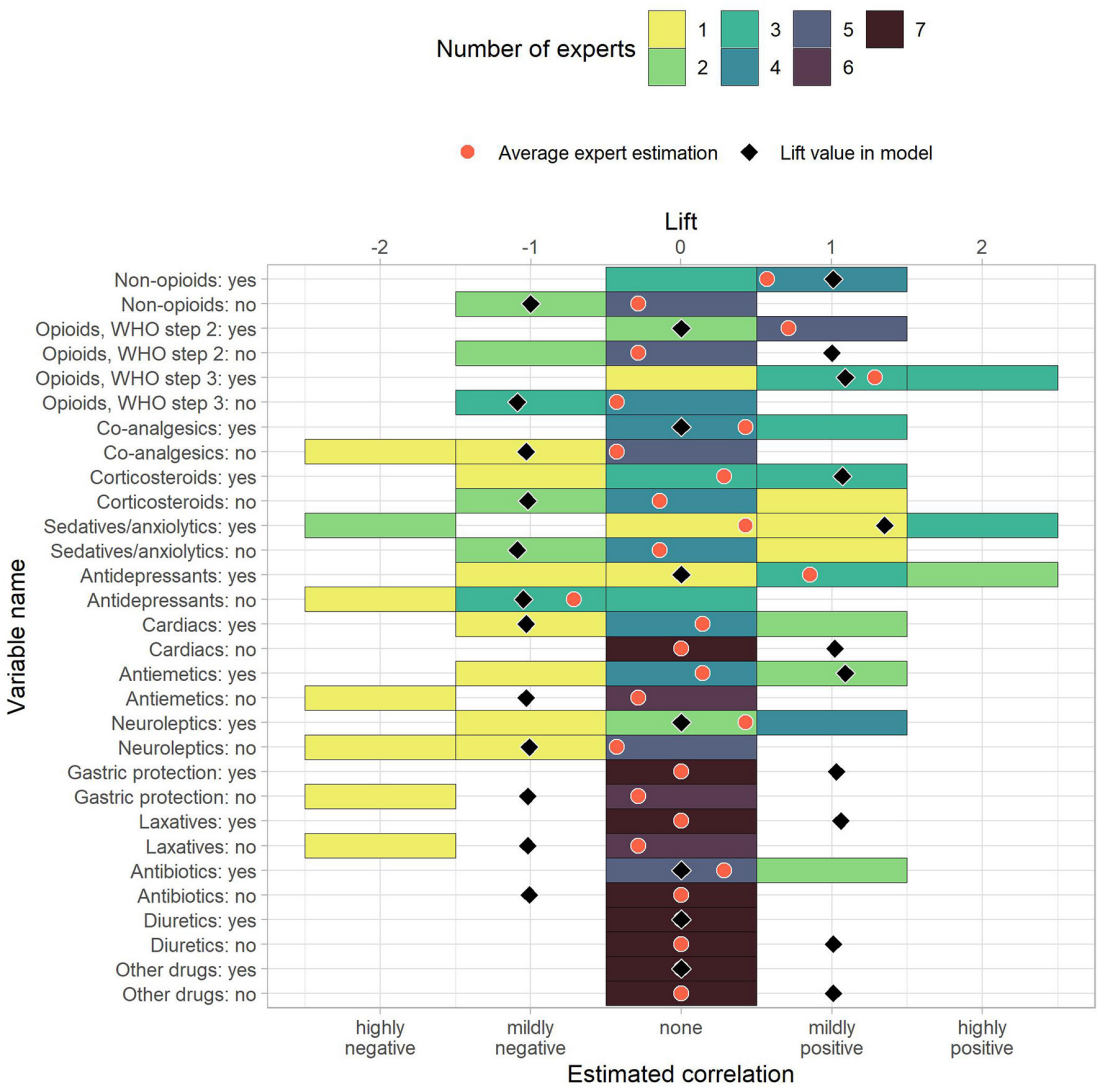
The estimated correlations of HOPE variables to anxiety for drug uptake variables. The color indicates how many experts chose the corresponding option. The black diamond indicate the lift value of the corresponding variable in the model, where 0 stands for “not part of the model” and negative values correspond to the lift of no-rules. The red dot is the average expert estimation.

**Figure 5:**
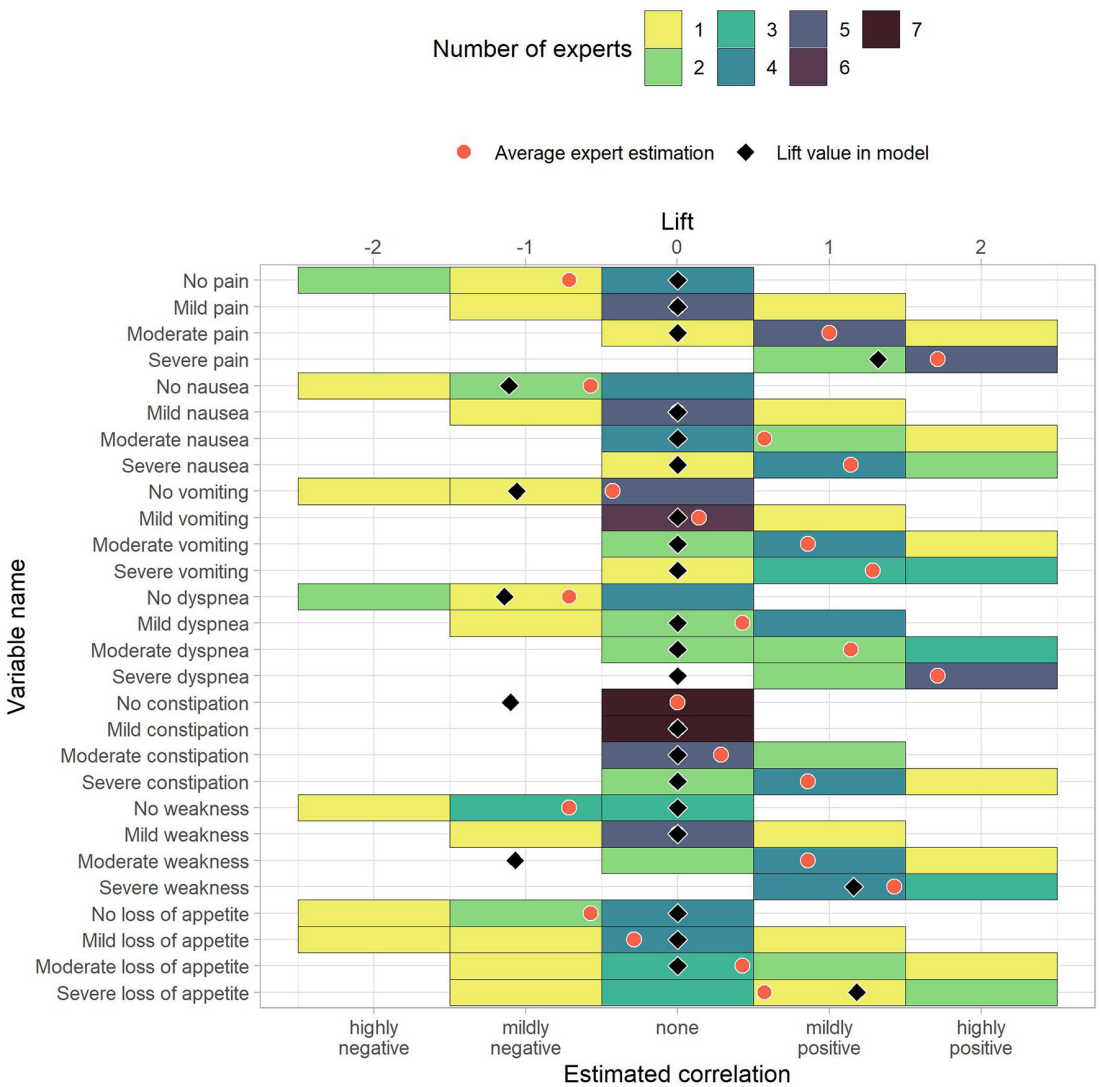
The estimated correlations of HOPE variables to anxiety for the patient's status (first part). The color indicates how many experts chose the corresponding option. The black diamond indicate the lift value of the corresponding variable in the model, where 0 stands for “not part of the model” and negative values correspond to the lift of no-rules. The red dot is the average expert estimation.

**Figure 6:**
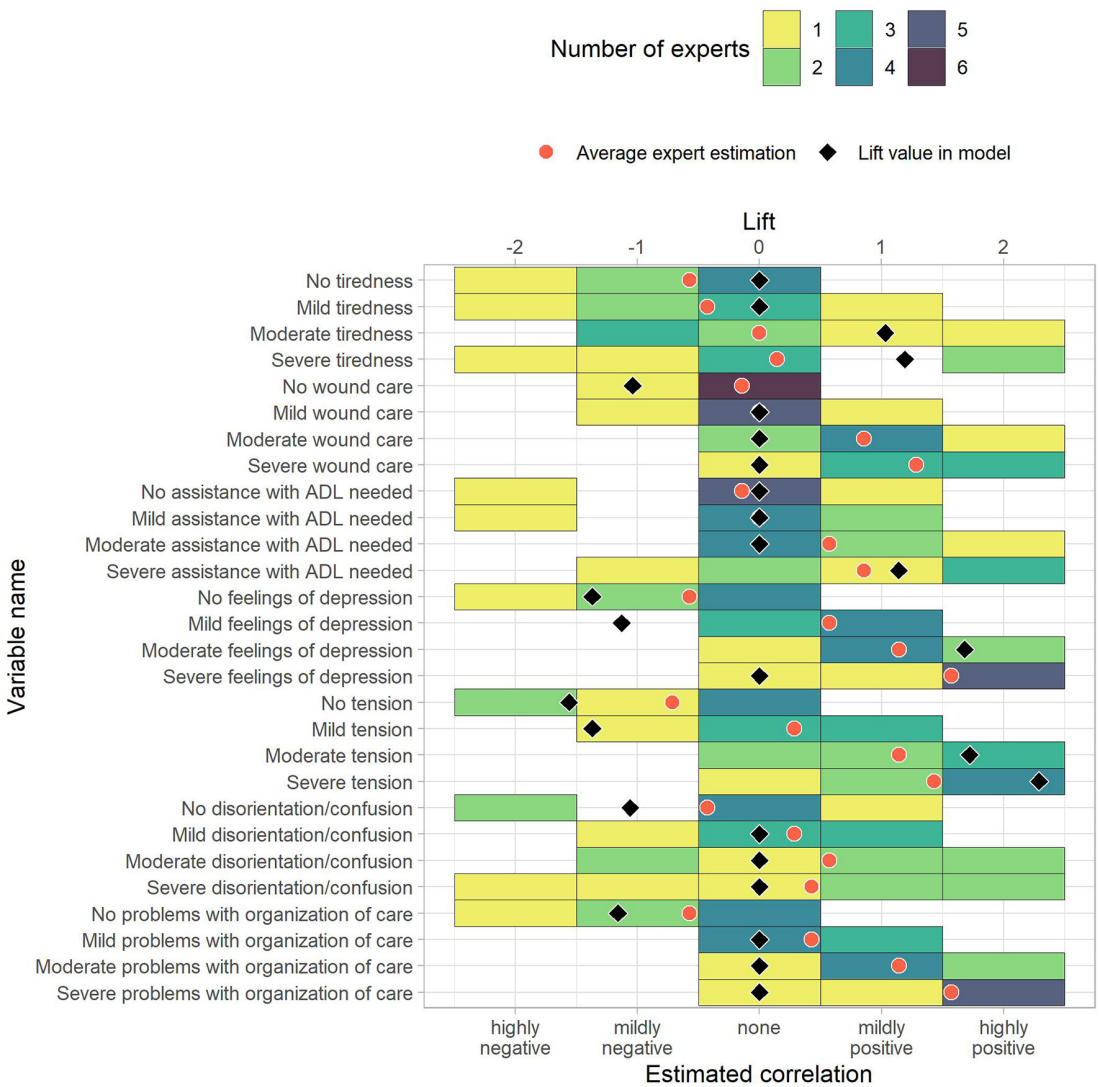
The estimated correlations of HOPE variables to anxiety for the patient's status (second part). The color indicates how many experts chose the corresponding option. The black diamond indicate the lift value of the corresponding variable in the model, where 0 stands for “not part of the model” and negative values correspond to the lift of no-rules. The red dot is the average expert estimation.

We received and evaluated seven questionnaire responses from palliative care professionals. Due to the low number of responses, these results are considered preliminary. The questionnaire responses are shown together with the corresponding prediction model lift values in Figures 3–6. To ease the comparison, lift values were considered negative for no-rules, as the correlation with “no anxiety” is also negative.

To measure similarity, we computed differences between the average correlation estimate across experts and the prediction model lift vales. The average absolute differences between average expert rating and prediction model were 0.70. Out of 118 items, ten had a difference of 0.0, and 86 showed an absolute difference of 0.75 or less. Of the remaining 32 items, the largest differences were reported for “Male” (−1.05 vs. 1.00), “Moderate weakness” (−1.07 vs. 0.86), “Severe dyspnea” (0.00 vs. 1.71), “Mild feelings of depression” (−1.13 vs. 0.57), and “Mild tension” (−1.37 vs. 0.29). These pairs can serve as incentives for future studies that further analyze the association to anxiety in palliative patients. The rules in the Supplementary Materials also include the average correlation given by the experts.

Apart from the item rating, five of the professionals reported a correlation between anxiety and some diagnosis groups. Other variables that were mentioned as potentially correlated with anxiety included chemotherapy and immunotherapy drugs as well as psycho-social factors, which are currently not part of HOPE.

## 5. Discussion

Earlier detection of anxiety and high risk of anxiety-creating conditions can lead to earlier treatment. In this work, we proposed a Bayesian-inspired rule mining approach to identify conditions in routine care that link to anxiety.

Previous work of Hofmann et al. on classifying anxiety in the HOPE data set achieved an AUC of 0.72 (34). Hofmann et al. employed multivariate logistic regression. Variables were analyzed for statistically significant interrelation with anxiety, which resulted in 15 of the 39 variables (excluding anxiety) being included in the regression. Non-linear transformations were used to map the result into a valid probability space of [0,1]. In comparison to Hofmann et al., our IBC rule mining approach yielded an AUC increase by 23.6%. A one-sided DeLong's test (47) was used to assert that our model significantly outperforms the state of the art (*p* < 0.001). In addition to the quantitative improvement, our approach offers better interpretability. Due to the non-linear transformations in Hofmann et al., variable influence on predictions are difficult to separate. In contrast, the mined rules offer convenient mapping into textual form that can explain the algorithm's reasoning. In addition, our methodology, with an 84% accuracy, outperforms results obtained in informal explorations (32) where nurses were able to detect anxiety with a 74% accuracy. In palliative care, and to our knowledge, anxiety prediction using clinical data has not been widely investigated. There is significant research on understanding anxiety's effect on the patient's quality of life and different methodologies using questionnaires/interviews to detect it, as summarized by Hofmann (35). But, we were unable to find other methods that use clinical data to detect anxiety.

In comparison to other widely used Machine Learning models, methods based on association rules are easy to interpret due to the easy-to-understand nature of association rules and the simple structure of the model. The rules can be used to analyze the underlying training data set, explicitly revealing the connections between the variables in the data set and the target variable. Furthermore, association rules can be directly compared to expert knowledge, simplifying the cost for conveying model knowledge to experts. Other, less interpretable models can be analyzed using *model-agnostic methods*, which build upon any model and derive explanations on the model's decisions (48). Examples of such models include LIME (49) and SHAP (50). While these methods make existing black-box models more transparent to end-users, they show some limitations, such as multiple explanations being in general inconsistent with each other, the inherently approximate nature of such explanations, and the separation of the model's explanation from the model itself (51). These limitations may have concrete consequences, such as loss of users' trust or the inability to add user feedback into the model (51). This is why we focus on association rules, which are easily explainable without these limitations.

The proposed method is subject to a form of overfitting with respect to the maximal rule length *r*_*l*_. In general, the AUC drops if longer rules are allowed, implying that these rules, which are derived from the training set, do not apply to the test. Another interpretation of the overfitting phenomenon is related to the partitioning of the space created by more complex rules. As *r*_*l*_ increases, each added item to the premise creates a partition that selects less rules than before. By sampling the entire data set to create the test and training sets, there are no guaranties that the partition was sampled fairly. Thus, biases are created by selected rules that otherwise would have been ignored.

The comparison of rounded lift values to experts' opinions allowed us to differentiate expected (86 out of 118) from unexpected (32 out of 118) items. Unexpected rules might serve as starting points for future studies to better understand their effect or the underlying confounding factors with respect to anxiety. The differences between lift and average expert opinion is merely a measure of relative similarity and not a comparison of lift values, i.e., the differences have no inherent meaning and can only be compared to each other and not to the original values. The experts disagreed on the correlations with anxiety in most items. In only 11 out of 118 items, all experts agreed. Four of the items, where experts agreed, matched with the prediction model. There were also major differences between the experts' opinions and the prediction model, indicating that the variable effects on anxiety (or confounding factors that affect both the variables and anxiety) are either hard to decipher or unknown. The presented method offers an objective way of assessing variables and their correlation with anxiety.

The experts' opinions allow us to study which model associations are expected and which are unexpected to care providers. For example, in the case of the association of gender and anxiety, studies have shown that female palliative patients are more likely to develop anxiety (52, 53), which is supported by our model, but expert opinion seems to suggest that the perceived correlation does not warrant adapting their daily clinical work. However, the questioned palliative care professionals expected a positive correlation between being male and developing anxiety, while the rule is part of the negative associations with anxiety in the model. The difference was the highest between model and expert opinion. Four of the professionals expected a (mildly or highly) positive correlation, while three experts expected no correlation. The discrepancy can be explained in two different ways. First, it might be caused by male patients being less direct about their emotional state than female patients. Second, the professionals could be overestimating the male patients' tendency to develop anxiety in palliative care. Future research is needed to come to a conclusion.

Use of sedatives and anxiolytics form one combined variable in HOPE making it challenging to analyze the true association between anxiolytics and anxiety. Use of sedatives or anxiolytics was reported for 1,302 patients without anxiety and 1,377 patients with anxiety, resulting in the associations “sedatives/anxiolytics: no” with no anxiety (lift 1.09, local support 79%) and “sedatives/anxiolytics: yes” with anxiety (1.35, 37%) being included in the model. The experts did not expect an association, with average opinions being −0.14 for “no” and 0.43 for “yes,” showing that the inclusion of sedatives and anxiolytics in the model is advantageous but is ultimately not as highly associated with anxiety as one could expect.

A common limitation of machine learning methods is their lack of interpretability. Our rule mining approach allowed us to make interpretable predictions. However, the rules cannot give causal explanations. Further research is needed to give an explanation for the unexpected associations, as in the example of male patients being negatively associated with anxiety while the palliative care experts expected a positive correlation.

While the proposed method was designed for binary classification, it can easily be generalized to multi-class classification by learning rules with each class on the right-hand side. The prediction can then be made by using the class of the rule that applies to the observation and has the highest lift as the prediction.

## 6. Conclusion

We proposed a novel method to predict anxiety in palliative patients based on the public HOPE data set. Our prediction model outperforms the current state of the art by 23.6% with an AUC of 0.89. The atomic rules mined from the data set provide deep insight into the variable relations and can be converted into text. By comparing experts' opinions on item correlations with our prediction model rules, we discovered several items that merit further investigation.

## Data Availability Statement

The data sets analyzed for this study can be found in the supplementary material in the study by Hofmann et al. (34) at https://journals.plos.org/plosone/article?id=10.1371/journal.pone.0179415.

## Author Contributions

LL and OH initiated the project, provided the idea for the method, discussed and validated the results. LL, OH, and TS wrote the manuscript and designed the questionnaire. SH provided the data set. OH programmed the method. All authors substantially revised the manuscript.

## Conflict of Interest

The authors declare that the research was conducted in the absence of any commercial or financial relationships that could be construed as a potential conflict of interest.

## Publisher's Note

All claims expressed in this article are solely those of the authors and do not necessarily represent those of their affiliated organizations, or those of the publisher, the editors and the reviewers. Any product that may be evaluated in this article, or claim that may be made by its manufacturer, is not guaranteed or endorsed by the publisher.
